# Predicting the Effects of Drug Combinations Using Probabilistic Matrix Factorization

**DOI:** 10.3389/fbinf.2021.708815

**Published:** 2021-08-13

**Authors:** Ron Nafshi, Timothy R. Lezon

**Affiliations:** Department of Computational and Systems Biology, University of Pittsburgh, Pittsburgh, PA, United States

**Keywords:** combination therapies, phenotypic screening, drug discovery, matrix factorization, active learning, experimental design

## Abstract

Drug development is costly and time-consuming, and developing novel practical strategies for creating more effective treatments is imperative. One possible solution is to prescribe drugs in combination. Synergistic drug combinations could allow lower doses of each constituent drug, reducing adverse reactions and drug resistance. However, it is not feasible to sufficiently test every combination of drugs for a given illness to determine promising synergistic combinations. Since there is a finite amount of time and resources available for finding synergistic combinations, a model that can identify synergistic combinations from a limited subset of all available combinations could accelerate development of therapeutics. By applying recommender algorithms, such as the low-rank matrix completion algorithm Probabilistic Matrix Factorization (PMF), it may be possible to identify synergistic combinations from partial information of the drug interactions. Here, we use PMF to predict the efficacy of two-drug combinations using the NCI ALMANAC, a robust collection of pairwise drug combinations of 104 FDA-approved anticancer drugs against 60 common cancer cell lines. We find that PMF is able predict drug combination efficacy with high accuracy from a limited set of combinations and is robust to changes in the individual training data. Moreover, we propose a new PMF-guided experimental design to detect all synergistic combinations without testing every combination.

## Introduction

Complex diseases are increasingly recognized as emerging not from single molecules, but from systemic dysfunction of biological processes. From a systems view, pharmacologically treating complex disease requires engaging multiple components of the dysregulated pathways through polypharmacology or combination therapies. Already combination therapies represent the standard of care for an array of diseases, including cancer (Mokhtari et al., 2017) bacterial infection (Mulani et al., 2019), HIV (Ghosn et al., 2018), neurological and behavior disorders (Ortiz-Orendain et al., 2017). They are also the focus of increasing attention in the search for therapeutics to treat other complex diseases, such as AD (Cummings et al., 2019) and NAFLD (Singh et al., 2017). Unfortunately, discovering effective combination therapies requires either serendipitous discovery in the clinic or laborious searches in pre-clinical models.

Phenotypic approaches have been more successful than target-based approaches in bringing new first-in-class drugs to the clinic (Swinney 2013, 2014), and next-generation *in vitro* disease models promise to boost the power of phenotypic screens by enhancing their clinical relevance. Screening in patient-derived 3D organoids (Lou and Leung, 2018; Takahashi, 2019) and biomimetic tissue chips that contain multiple interacting cell types in physiological geometries (Mittal et al., 2019; Gough et al., 2021), has proven useful for personalizing therapies for cancer and other complex diseases. Inter-organ side effects have been studied *in vitro* using multiple linked tissue chips (Skardal et al., 2017), paving the way for complete human-on-a-chip disease models. The price of this biological fidelity is a loss of throughput. Although sophisticated disease models more closely resemble their clinical counterparts than do their 2D monoculture predecessors, they are far more expensive to develop and maintain. Large-scale screens and even moderate combination screens are not yet feasible in these systems.

An addressable bottleneck in the phenotypic discovery pipeline is the low hit rate of screens. The overwhelming majority of compounds tested in traditional phenotypic screens are inactive, and the problem is exacerbated in combination screens, where the number of possible combinations scales exponentially with the size of the compound library. Fortunately, the availability of large screening data sets in human cell lines has spurred computational methods for predicting drug efficacies and synergies for complex diseases (Menden et al., 2019; Adam et al., 2020). Deep learning models using Graph Convolutional Networks are a powerful and well explored approach for relating complex relationships between inputs and targets, and many successful models have been developed for predicting values in the drug-interaction network (Sun et al., 2020). Other successful approaches include those that incorporate information on transcriptomic or proteomic profiling (Dawson and Carragher, 2014; Huang et al., 2019; Diaz et al., 2020), compound chemical structures (Sidorov et al., 2019), or drug targets (Yang et al., 2020; Rao et al., 2019; Iwata et al., 2015). Network-based approaches have been applied to create robust methods of identifying therapeutically effective drug combinations (Cheng, Kovács, and Barabási 2019).

Combination screens are amenable to statistical prediction methods that use screening data without supplementing it with details about chemical structures, targets, or OMICS profiles. One particularly powerful approach employs a higher-order factorization machine to predict dose-response surfaces for unique drug combinations using only data from the same screen (Julkunen et al., 2020). The advantages of such methods are that they guard against bias introduced from orthogonal databases, they can be used with unannotated compound libraries, and their predictions are consistent with the data generated in the screen of interest. For methods like this to be effective, the computational and experimental components of the screen must be properly synchronized, so that each informs the other (Stern et al., 2016). A number of studies have addressed the right way to mix computational and experimental work to find effective combination therapies (Calzolari et al., 2008; Gerlee et al., 2013; Kashif et al., 2015; Weiss et al., 2015; Silva et al., 2016; Matlock et al., 2017; Ianevski et al., 2019).

Here we introduce a method for predicting the effects of drug combinations using as input only the effects of other drug combinations. Mathematically, the problem is identical to filling in the missing values of a symmetric matrix containing the effects of drug combinations. Each row and each column of the matrix corresponds to a drug, and the matrix elements are the effects of the drug combinations. If only a subset of the matrix elements are known, the rest can be inferred by decomposing and reconstructing the partial matrix under certain assumptions (Lezon et al., 2006; Lezon and Bahar, 2010). For the current application, we use Probabilistic Matrix Factorization (PMF) (Salakhutdinov and Mnih, 2007), a collaborative filtering algorithm that has proven successful in other problems of the same class (for an introduction to collaborative filtering algorithms, see Aggarwal, 2016).

The PMF algorithm was first developed to recommend movies to Netflix users based on the movies viewed by other users. The core assumption of PMF is that attitudes or preferences that lead to each user’s score for a movie are shared by other users with similar taste. PMF recommends that viewers watch movies that similar viewers enjoyed. The method has since been applied to predict values from other large, sparse and imbalanced data sets. Biomedical applications of PMF include predicting diseases associated with transcription patterns (Ha et al., 2020; Mao, Wang, and Zhang 2019), recommending novel indications for drug repurposing (Meng et al., 2021; Yang et al., 2014), and predicting novel targets from drugs (Cobanoglu et al., 2013; Cobanoglu et al., 2015; Li et al., 2020). In the present context, PMF is used to “recommend” drug combinations based on the known effects of similar combinations. We train our model on phenotypic screening data from the NCI ALMANAC (Holbeck et al., 2017), a robust collection of pairwise drug combinations of 104 FDA approved anticancer drugs against 60 common cancer cell lines. We find that knowing the effects of only 50% of drug combinations allows us to classify the effects of the missing combinations as efficacious with 95% accuracy, and we demonstrate how our method can be incorporated into optimal experimental design.

## Methods

### NCI ALMANAC

The NCI ALMANAC is a novel, easy-to-use resource created to help researchers identify new combination therapies. The NCI ALMANAC database (Holbeck et al., 2017) is a collection of pairwise combinations of 104 FDA approved anticancer drugs against the NCI-60, a set of 60 common human tumor cancer cell lines collected by the National Cancer Institute. A total of 5,232 drug-drug pairs were evaluated in each of the cell lines; 304,549 experiments were performed to test each drug at either 9 or 15 combination dose points, for a total of 2,809,671 dose combinations. At each dose combination, the percent cell growth after 2 days was measured and recorded, and the efficacy of the combination calculated as the percent of growth inhibition. A combination that has no effect on cell growth compared to control has zero efficacy; a combination that completely halts cell growth has efficacy 100. See the NCI ALMANAC (Holbeck et al., 2017) for details. For each cell line, the combination efficacies are arranged into a symmetric matrix, $M_{104x104}$ , where each row and column represent a drug, and each element represents the efficacy of a unique drug-drug combination on that cell line. For purposes of PMF (see below), diagonal elements are ignored. The data is then normalized to mean-zero and unit variance for input into the PMF algorithm.

The synergy of each combination is reported by the NCI ALMANAC as a “ComboScore” that measures the difference between the recorded growth rate after testing and the growth rate expected by Bliss Independence (Bliss, 1939). A positive ComboScore indicates a synergistic combination, whereas a negative ComboScore indicates an antagonistic combination. When applying PMF to predict synergies instead of efficacies, we populate the input matrix **M** with ComboScores and normalize as described above.

### PMF

Probabilistic Matrix Factorization (PMF) is a collaborative filtering algorithm that factors the low-rank input matrix $M_{n\times m}$ into the product of two low-rank matrices, $A_{n\times d}$ and $B_{m\times d}$ such that $M_{\mathrm{ij}}=A_{i}B_{j}^{T}$ . Thus, PMF reduces to estimating the two matrices A and B . The core assumptions of this are that the values of M are independent, normally distributed and share a common variance $\sigma^{2}$ . Thus, the conditional probability of entries of M can be expressed as

$$p\left(M\text{|}A,B,\;\sigma^{2}\right)=\prod_{i=1}^{n}\prod_{j=1}^{m}N{\left(M_{\mathrm{ij}}\text{|}A_{i}B_{j}^{T},\;\sigma^{2}\right)}^{I_{\mathrm{ij}}}$$

where $I_{\mathrm{ij}}$ is the indicator function equal to 1 if $M_{\mathrm{ij}}$ is known and 0 otherwise (Salakhutdinov and Mnih, 2007).

To solve for the matrices **A** and **B**, we place a zero-mean spherical Gaussian prior on each, such that $p\left(A|\sigma_{A}\right)=\overset{N}{\underset{i=1}{\prod }}N\left(A_{i}\text{|}0,\sigma_{A}^{2}I\right)\;$ and $p\left(B|\sigma_{B}\right)=\overset{N}{\underset{i=1}{\prod }}N\left(B_{i}\text{|}0,\sigma_{B}^{2}I\right)$ . We can then derive the full posterior distribution of **A** and **B** as $p\left(A,B\text{|}M,\sigma^{2},\sigma_{A}^{2},\sigma_{B}^{2}\right)\propto p\left(M\text{|}A,B,\;\sigma^{2}\right)p\left(A|\sigma_{A}^{2}\right)p\left(B|\sigma_{B}^{2}\right)$ . Maximizing the log-posterior is equivalent to minimizing the sum-of-squared-errors objective function: $L\left(A,B\right)=\frac{1}{2}\overset{N}{\underset{i=1}{\sum }}\overset{M}{\underset{i=1}{\sum }}I_{ij}{\left(M_{\mathrm{ij}}-A_{i}B_{j}^{T}\right)}^{2}+\frac{\lambda }{2}\overset{N}{\underset{i=1}{\sum }}||A_{i}|{|}_{\mathrm{Fro}}^{2}+\frac{\lambda }{2}\overset{M}{\underset{j=1}{\sum }}||B_{j}|{|}_{\mathrm{Fro}}^{2}$ , where λ is the regularization rate hyperparameter. We then construct a stochastic gradient descent update scheme by differentiating the loss function in terms of **A** and **B**, such that

$$\frac{\partial }{\partial A_{i}}L\left(A,B\right)=\sum_{j=1}^{M}I_{ij}\left(M_{\mathrm{ij}}-A_{i}B_{j}^{T}\right)B_{j}+\lambda A_{i}$$

$$\frac{\partial }{\partial B_{j}}L\left(A,B\right)=\sum_{j=1}^{N}I_{\mathrm{ij}}\left(M_{\mathrm{ij}}-A_{i}B_{j}^{T}\right)A_{i}+\lambda B_{i}$$

Algorithmically, we randomly initialize **A** and **B** from Gaussian distributions and iteratively update them by descending along these gradients until a minimum of L(A,B) is reached. Applying these rules simultaneously to both **A** and **B** guarantees convergence of the algorithm to a local minimum. However, the stochastic nature of the initial conditions implies that each run of PMF may not necessarily converge to the global minimum, or even the same local minimum. This requires that PMF be run multiple times on different random initializations and then select the most accurate factorization. While this increases the overall computational cost, this is offset by PMF’s computational cost scaling linearly with input size and using lightweight low-rank approximations.

Stochastic gradient descent methods are a critical component of machine learning, and methods incorporating momentum and acceleration play an important role when used in conjunction with stochastic gradients (Assran and Rabbat, 2020). Momentum methods help accelerate stochastic gradient descent in the relevant direction and dampen oscillations as a minimum is approached by incorporating the momentum constant γ . The update step with respect to the parameters θ can be expressed as $v_{t}=\gamma v_{t-1}+\eta \nabla_{\theta }J\left(\theta \right),\;\;\theta =\theta -v_{t}$ . However, simple momentum methods can be insufficient for complex surfaces. The Nesterov Accelerated Gradient (NAG) (Assran and Rabbat, 2020) improves on this method by “looking ahead” to where the parameters will be to calculate the gradient and is formalized as follows: $v_{t}=\gamma v_{t-1}+\eta \nabla_{\theta }J\left(\theta -\gamma v_{t-1}\right),\;\;\;\theta =\theta -v_{t}$ . Rather than computing the gradient at parameters θ , NAG looks ahead at a rough approximation of where the parameters will be, computing the gradient at $\theta -\gamma v_{t-1}$ . This anticipatory update greatly increases optimization and performance of PMF as it approaches a minimum.

## Results

### PMF Accurately Recovers Drug Synergies From Partial Data

**FIGURE 1:**
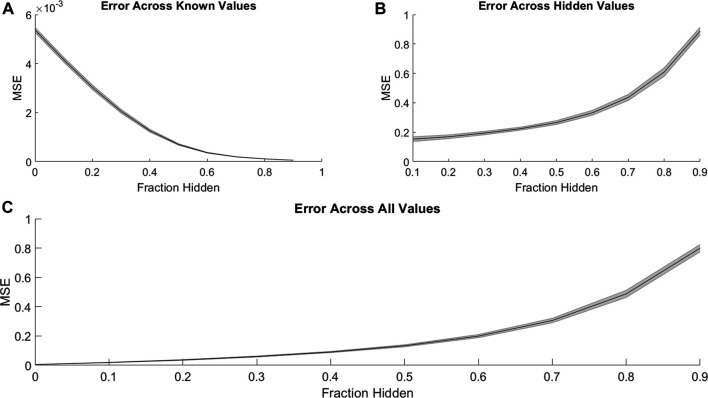
PMF recovers the values of hidden elements of the drug efficacy matrix from only a fraction of interactions. The mean-squared error of PMF in recovering values of **(A)** known, **(B)** hidden, and **(C)** all elements is plotted against the fraction of hidden data. In all panels, the shaded area represents the standard deviation of the mean-squared error over 25 trials across all cell lines.

**FIGURE 2:**
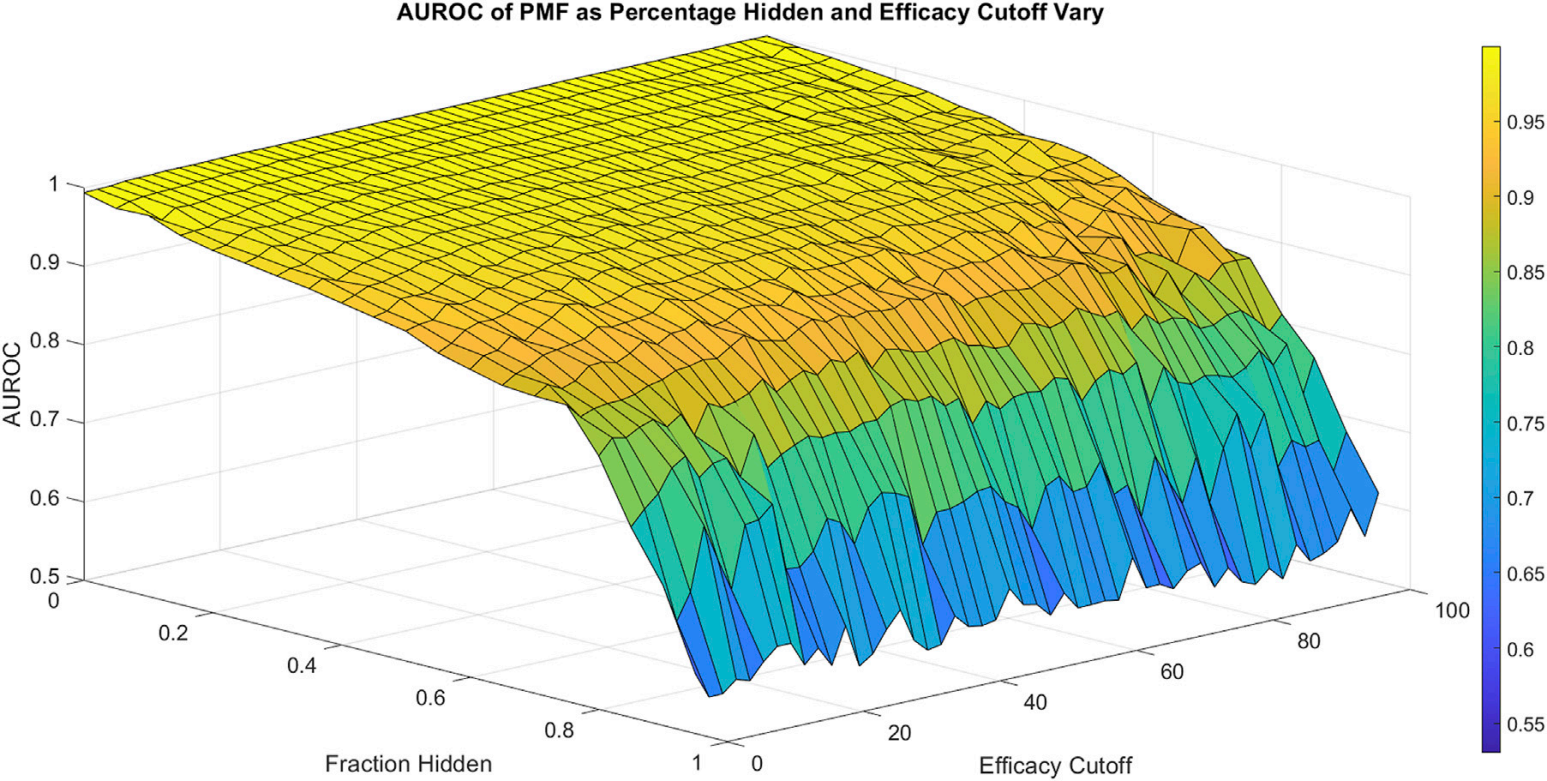
The area under the ROC curve (AUROC) of PMF is shown as the fraction hidden and efficacy cutoff vary on the 786-0 cell line, which is representative of all cell lines. The efficacy cutoff describes the efficacy at which a drug-drug combination is considered active, with combination efficacy defined as 100 minus the percent growth as described in the standard NCI-60 testing protocol (Holbeck et al., 2017). As the fraction hidden decreases, the performance of the model remains high until it drops sharply at 70% hidden and performs with similar accuracy regardless of the efficacy cutoff, decaying to random guesses when the full matrix is hidden. The smooth surface indicates PMF reproduces all elements with equal accuracy and is not heavily affected by outliers.

We first investigated the ability of PMF to recover hidden elements in the drug combination efficacy matrix. For each cell line, we randomly hid a fraction of the combination efficacy matrix, creating non-overlapping “training” and “validation” sets. Then, we used PMF to predict the hidden values and complete the matrix. To guarantee a solution, we included only cases where all drugs were present in a single connected component; that is, where a path could be made from any drug to any other drug using common combination partners. PMF recovered training data to arbitrary precision (Figure 1A) and recovered test data well, provided a sufficiently large training set (i.e., small fraction of data hidden). Using empirically determined hyperparameters for the regularization rate (λ), learning rate ( η ), and momentum constant (γ), we found that knowing only 30–50% of the drug-drug interactions was sufficient to recover the remaining values in the matrix to within 10% (Figures 1B,C). When selecting combinations with efficacies above a given threshold, PMF performance did not vary strongly with the threshold value (Figure 2); that is, the method can predict whether a combination has an effect over 0.9 nearly as well as it can predict whether a combination has an effect over 0.2.

### PMF Performance Is Largely Independent of Individual Drug Efficacies

Assuming compounds act independently (i.e., Bliss independence), the most efficacious compound combinations will be combinations of the independently most efficacious compounds. Reasoning that efficacious drugs are more likely to influence pathologically relevant mechanisms, we next investigated whether PMF performed better when trained on combinations involving highly efficacious drugs. For each cell line, we rank-ordered the compounds by efficacy and then divided the drug efficacy matrix into halves and quarters to assess if PMF is more accurate when only provided singularly efficacious drugs.

On aggregate we find the differences in accuracy were small, and PMF performance was largely independent of the individual efficacies of the starting set outside of this edge case (Supplementary Figures 1A,B). More generally, we found that the most efficacious compounds neither led to the most efficacious combinations, nor were they the best at predicting the values of missing efficacies (Supplementary Figure 1C). In fact, individual drug identities did not greatly affect the accuracy of the prediction. We generated an occupancy matrix by randomly selecting 10% of the elements in the combination efficacy matrix. We then randomly shuffled the identities of the drugs while keeping the occupancy matrix static. Repeating this 1,000 times for 1,000 different occupancy matrices, we found PMF predicted the missing values of each matrix with a mean squared error of 0.938 ± 0.0145, and thus performed equally well regardless of the individual drug identities for a given occupancy matrix.

### Graph Topology’s Influence on PMF Performance

Viewing the problem though a graph lens, the combination efficacy matrix describes an undirected graph in which the *N* drugs are nodes and known two-drug combinations represent weighted edges (for a primer on network science, see Barabási, 2016). The challenge of PMF is to reconstruct a fully connected graph from a seed network. By using different algorithms for selecting drug combinations for the training set, we investigated how seed network topology influences prediction accuracy. The method described above, where seed drug combinations are selected randomly and independently, is known as an Erdős-Réyni graph (Rényi and Erdős, 1959) that has a Poisson degree distribution (Barabási and Pósfai 2016).

An extension of the Erdős-Réyni graph is the Watts-Strogatz model (Watts and Strogatz, 1998). This method is motivated by the observation that often in real networks, almost any node can be reached by a short number of steps, known as the Small-World Property (Milgram, 1967). The Watts-Strogatz graph is generated by attaching each node to its nearest k neighbors, resulting in a regular lattice structure. Each edge is then randomly reassigned with probability β. When β is 0, no changes are accepted, and the method preserves the original lattice. As β increases, more links will be randomly assigned, and as β approaches 1, all links will be randomly reassigned, resulting in a completely random Erdős-Réyni network. Intermediate values of β result in small-world networks of low diameter (Barabási and Pósfai 2016).

**FIGURE 3:**
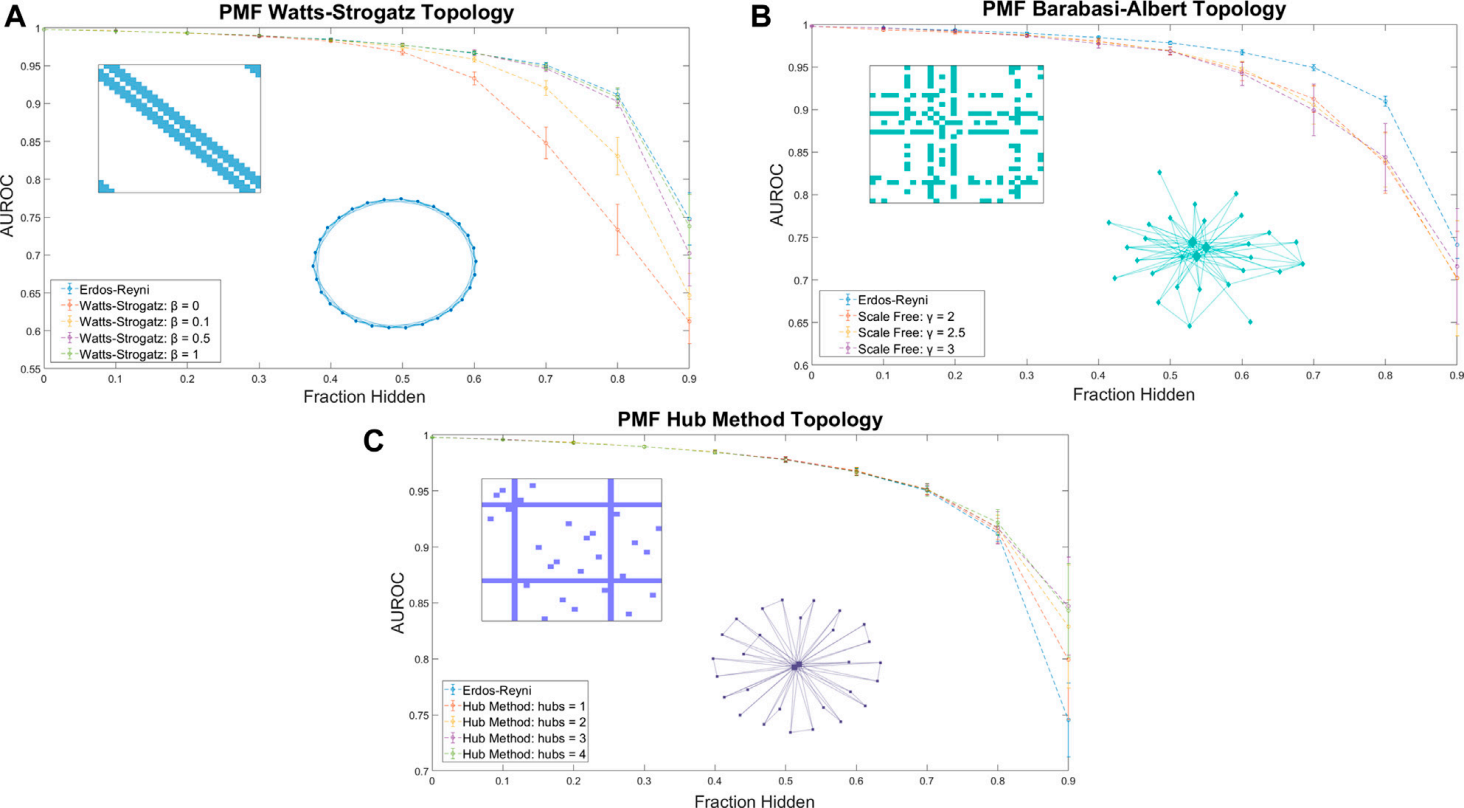
The AUROC of PMF in identifying efficacious combinations as the fraction of the data hidden increases is measured for **(A)** Watts-Strogatz graphs, **(B)** Barabási-Albert scale-free graphs, and **(C)** graphs generated by the Hub Method. Included in each plot is a sample of the adjacency matrix and topology of each network. Error bars represent standard deviation over 25 repeated trials at the same value of β. **(A)** Watts-Strogatz graphs with varying β. When β is near zero, each drug has *k* connections with its nearest neighbors in a lattice structure, and the model performs worse than reproducing from an Erdős-Réyni distribution of equivalent size. As β approaches 1 and the degree distribution of the graph converges to a similar Poisson Distribution of an Erdős-Réyni graph, the accuracy of the predictions begins to approach the level of accuracy seen with purely random topologies. **(B)** Scale-free seed networks perform similarly, regardless of scaling exponent. On aggregate, scale-free graphs perform similarly for all values of γ, and slightly underperform compared to Erdős-Réyni topologies. **(C)** Graphs generated using the hub method with random hubs produce more accurate predictions than other graph types. When most data are hidden, error and standard deviation of the prediction decrease as the number of hubs increases.

When training data was arranged in a Watts-Strogatz model topology, the performance of PMF increased with β (Figure 3A). We attribute the poor performance near β = 0 to the difficulty of predicting combination effects of drugs that are separated by large distances on the seed network. The adjacency matrix for a regular lattice is banded, with the unknown values comprising a contiguous block. Performance improves for values of β near ½, where the small-world property emerges, and peaks at β = 1, the Erdős-Réyni network. Whereas the small-world Watts-Strogatz graph provides a short path between any pair of nodes, the Erdős-Réyni graph contains multiple paths, each carrying evidence for the value of the inferred combination efficacy.

Many real-world networks do not follow a binomial or Poisson degree distribution, and instead follow a power law or scale-free distribution. In a scale-free network, the probability that a node has *k* edges is proportional to *k* ^−γ^, where γ is a scaling exponent between 2 and 3. We explored whether a scale-free distribution in the input data influences the accuracy of the prediction. Using the hidden parameter model (Caldarelli et al., 2002; Söderberg, 2002; Boguñá and Pastor-Satorras, 2003), we generated scale-free seed networks for training PMF. The method performed equally well for scale-free distributions for all values of γ, and predicted unknown values with accuracy comparable to the Watts-Strogatz method (Figure 3B).

Designing a combination screen using the above-described graph topologies may not be experimentally convenient; instead, screeners are more likely to select a few well-known compounds and test them in combination with other compounds in a large library. The adjacency matrix of the seed graph in this approach has several rows/columns in which every value is known, while the large majority of have few or no known values (Figure 3C). The corresponding graph has several fully connected hubs, with the remaining nodes having very few connections.

We explored the accuracy of PMF by using a hub method construction defined as follows: First, we ensured that every node had exactly one connection. Then, we selected nodes at random to be hubs, and ensured that each hub was fully connected. Finally, the remaining edges were randomly assigned in an Erdős-Réyni random fashion. We found the PMF performed stronger on hub method topologies than random Erdős-Réyni topologies when more than 80% of the network was hidden and the graph was sparse. Moreover, when training data was arranged in a hub model topology, the performance of PMF increased as the number of hubs increased (Figure 3C).

Thus, we found that the specific seed topology of the training data did not greatly affect the accuracy of the prediction in identifying synergistic drugs if the topology was a random Erdős-Réyni graph or had a binomial degree distribution, such as the Watts-Strogatz for large β. However, PMF did perform worse when edges were evenly distributed following the Watts-Strogatz model for small values of β or when edges were distributed following a scale-free distribution. Moreover, we found that PMF was more accurate under hub topologies mirroring real drug combination assays when more than 80% of the network was hidden, which is exactly the region of interest if we want to test as few combinations as possible.

### PMF Predicts Efficacy, But Not Synergy

The desired output of most phenotypic combination screens is an efficacious and non-toxic combination; however, *de novo* development of combination therapeutics will benefit from identifying synergistic drug combinations, whether or not they are efficacious. For example, two drugs that individually have no efficacy may have a moderate effect in combination. Although such a combination may not be clinically useful, it carries structure and pathway information that may serve as the starting point for rational development of combination therapeutics.

**FIGURE 4:**
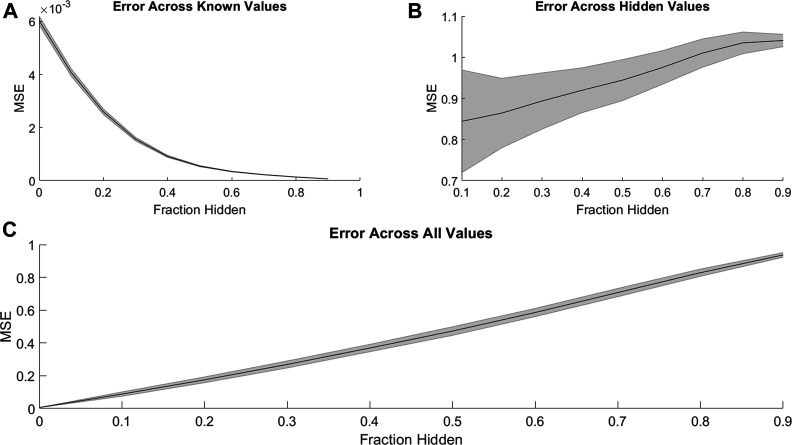
Similar to Figure 1, the mean-squared error of PMF in recovering combination synergy of **(A)** known, **(B)** hidden, and **(C)** all elements are plotted against the fraction of hidden data. In all panels, the shaded area represents the standard deviation of the mean-squared error over 25 trials across all cell lines. Once again, PMF recovers all known data to arbitrary precision. PMF performs with much less accuracy when predicting ComboScores rather than efficacies (Figure 1). Error is much greater and more uncertain overall in hidden indices and thus across all indices.

PMF was able to recover missing values much less accurately when predicting synergy rather than efficacy. Just as with efficacies (Figure 1), PMF recovered training data to arbitrary precision (Figure 4A), but it did not recover test data well, unless it had a sufficiently large training set (i.e., small fraction of data hidden) (Figures 4B,C). While the accuracy of PMF on predicting synergy was much weaker than PMF predicting efficacy, we still found that the model is robust and performed well in cases where 50–70% of the total matrix was known.

This stark decrease in accuracy and predictive power may result from the lopsided definition of synergy. The ComboScore of each drug combination represents the difference between the observed effect of the combination and the expected effect assuming each drug acts independently. Because the upper bound on efficacy is the same for individual drugs and combinations, a combination of highly efficacious drugs cannot have a high ComboScore, even if it has optimal efficacy. Similarly, combinations with identical efficacies may have different ComboScores, depending on the efficacies of the individual drugs used in the combinations. Thus, a low ComboScore reveals nothing about the efficacy of the combination, but a high ComboScore indicates the combination’s component drugs individually have low efficacy (Supplementary Figure 2). As ComboScores are calculated from individual and combination efficacies, one can still use PMF to predict combination efficacies, and use these to calculate ComboScores.

### PMF as a Tool to Guide Combination Screening

*In vitro* phenotypic-based screens have several benefits for drug discovery, such as not needing to know the molecular target of a disease and being less restricted by hypotheses (Zheng et al., 2013). However, throughput can be low in such assays, and increasing the number of compounds to be screened causes experimental effort and cost to rise exponentially. PMF may help combat this issue by guiding combination screens through iterative prediction and testing in an active learning scheme.

We simulated PMF being used in an active learning experimental design as follows. First, we created a random Erdős-Réyni graph topology with 10% of the total combinations known. Then, we used PMF to reproduce the entire combination efficacy matrix and identified the top 5% greatest efficacies as predicted by PMF. We then “tested” these identified efficacious combinations by adding the actual values of the efficacies to the list of known combinations, and then repeated the procedure to discover the next 5%, until the entire matrix is recovered.

**FIGURE 5:**
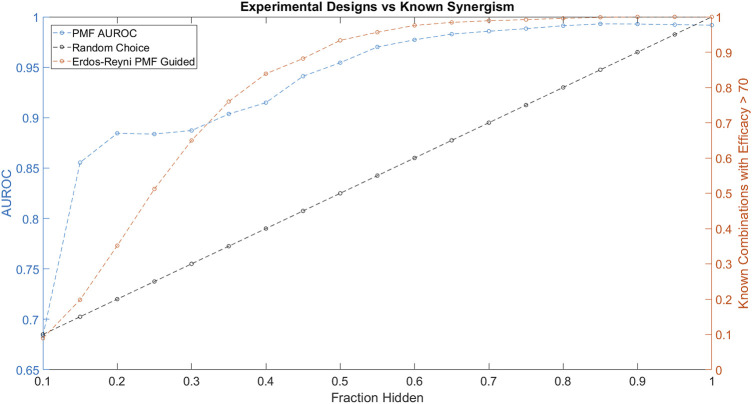
The performance of PMF in a proposed experimental design to predict drug combinations with efficacy greater than 70 is plotted in orange and is compared against random choice plotted in black. Both the AUC of PMF’s predictions in blue as well as the percentage of known efficacious drugs with efficacy greater than 70 are plotted against the known fraction of the drug-drug efficacy matrix. The experiment following random choice takes a random sample of the graph, resulting in a linear relationship between the amount of the drug-synergy matrix known and the amount of known synergistic drugs. As the procedure described above is repeated, PMF identifies more than 95% of the most efficacious combinations while only knowing 50% of the full drug efficacy matrix, much greater than random choice.

PMF-guided screens identified efficacious combinations much more efficiently than naïve random tests (Figure 5). In our simulated experiment, PMF identified efficacious combinations at three times the rate of random choice and identified as much as 95% of all highly efficacious combinations while only testing 50% of all available combinations. This finding was consistent across cell lines and was not sensitive to the details of the starting point. Our results suggest that screeners may be able to test a small number of relevant combinations of direct interest and obtain the remaining synergistic combinations following a PMF-guided design. Future studies could fruitfully explore this issue further by optimizing PMF-guided screens as well as investigating its accuracy applied in a physical assay experiment.

Throughout the simulated experiment, we monitored the performance of PMF as measured by AUROC (Figure 5). The dip in AUROC observed around the fourth step of the simulated experiment may be due to bias introduced by the active learning. Efficacious combinations are not uniformly distributed across all drugs, and indeed a small subset of drugs is likely to contribute to many of the efficacious combinations. As the experiment progresses, PMF preferentially selects combinations from an efficacious minority of the nodes, mirroring the construction of a scale-free graph. PMF performs worse on scale-free graphs compared to Erdős-Réyni graphs (Figure 3), causing the accuracy to decrease as nodes are preferentially tested, and then increase as these nodes are saturated and the rest of the matrix is tested. Future studies might investigate ways to counteract this drop in error by using a more complex method than simply testing the top 5% most efficacious combinations as predicted by PMF.

### The Method’s Performance Is Not Unique to Cancer

To test the performance of our method in diseases other than cancer, we applied it to data from a small combination screen for Huntington’s disease (HD), an autosomal dominant neurodegenerative disease caused by an abnormally long polyglutamine stretch in the huntingin protein (Zuccato et al., 2010). The clinical progression of HD starts with general loss of motor control around the third decade of life. This is followed by mood and personality changes, and eventual dementia and death. To date, there are no drug-like molecules that can prevent or slow HD, and the pleiotropic nature of huntingtin makes it difficult to target directly.

Pei and coworkers reported results from a combination screen in a murine cell-based model of HD (Pei et al., 2017). Briefly, their assay used serum deprivation to induce stress in neuronal progenitor cells derived from the ST*Hdh* ^*Q111*^ murine cell line model of HD, which has an abnormally long glutamine stretch in its huntingtin protein. The phenotypic response of the cells was then compared to that in serum-deprived cells of their isogenic wild type ST*Hdh* ^*Q7*^; overall, serum deprivation killed about half of the HD cells and about 5% of the wild type cells. The group evaluated 268 two-compound combinations of 32 compounds for their ability to protect against cell death. As a metric, they used “Percent Recovery,” which captures the percent of HD cells that were rescued from cell death by a treatment (Pei et al., 2017).

**FIGURE 6:**
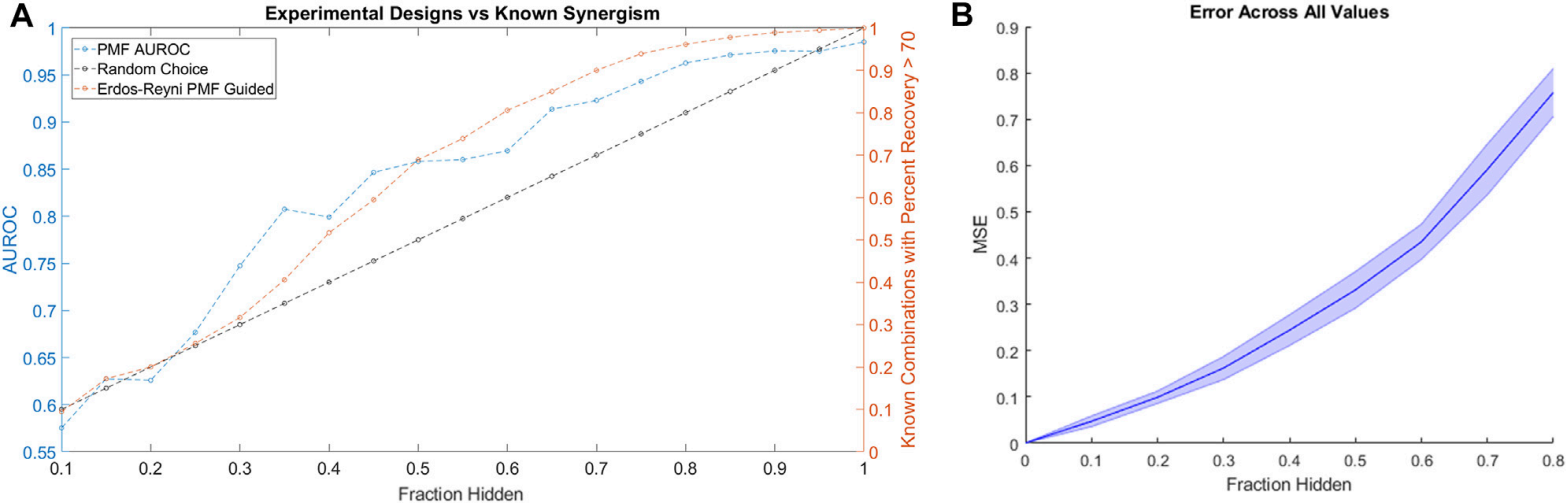
**(A)** The performance of a simulated PMF guided drug screen in HD identifies all drug combinations with Percent Recovery greater than 70. Both the AUC of PMF’s predictions (in blue) as well as the percentage of known efficacious drugs with efficacy greater than 70 (orange and black) are plotted against the known fraction of the drug-drug efficacy matrix. Once again, PMF guided screens outperform the rate of chance, with PMF identifying more than 90% of the most efficacious combinations while only knowing 70% of the full drug efficacy matrix. **(B)** The error of PMF in predicting all elements is plotted against the fraction hidden. PMF does as well on the smaller HD dataset as it does on smaller subsets of the ALMANAC data (Supplementary Figure 1B).

Applying the same simulated experimental design to the HD data as we applied to the ALMANAC data, we found that PMF-guided screens identified combinations with the highest Percent Recovery much more efficiently than the rate of random chance: Over 90% of combinations with Percent Recovery over 70 were identified by testing only 70% of all available combinations (Figure 6A). Moreover, we found that PMF performed with nearly the same accuracy on the HD dataset as it did on similarly sized subsets of the ALMANAC data (Figure 6B, Supplementary Figure 1B). While the guided screen is more efficient than naïve guessing, the results are much weaker than on the much larger ALMANAC dataset. Starting with 10% of the 32 compound combinations known, PMF may only know a handful of combinations, and struggles until around 30% of all combinations are known.

## Discussion

Our results show that it is possible to use information on the effects of drug combinations to predict the effects of novel combinations. A strength of our approach is that does not require any outside knowledge of chemical structures, target profiles, or OMICS data. This lack of reliance on outside data contributes to the method’s robust performance between data sets of similar sizes. Indeed, freedom from additional information augments our method’s stability and flexibility: Rather than predicting the effects of combinations of drugs, it could be used for combinations of unknown substances, natural extracts, or even combinations of combinations. As such, our method may contribute to identifying mechanisms of action for novel compounds. Since PMF is lightweight and is not informed by structure or other data, it may provide a benchmark against which more complicated methods can be tested.

There are many constrained low-rank matrix approximation algorithms that could have been used other than PMF. Within this general framework, some powerful techniques include singular value decomposition (SVD) (Golub and Reinsch, 1971), principal component analysis (PCA) (Wold et al., 1987), non-negative matrix factorization (NMF) (Lee and Sebastian Seung, 1999), entropy maximization (Lezon et al., 2006; Lezon and Bahar, 2010), and deep matrix factorization (MF) (Pierre and Siebert, 2020). SVD’s matrix factorization is unique and orthogonal unlike PMF. However, SVD’s matrix reconstruction is a superposition of the orthogonal components with arbitrary signs, losing strong correlations that may exist and the interpretability of the latent factor representation of PMF. Moreover, SVD has been shown to be less accurate than PMF (Salakhutdinov and Mnih, 2007), and is vulnerable to overfitting. PCA’s orthogonal decomposition suffers from similar weaknesses. The parts-based decomposition of NMF has provided powerful, interpretable methods for matrix factorization in a variety of applications from text mining to gene expression. However, NMF is poorly suited to drug discovery due to its non-negativity constraint. Methods relaxing this constraint (Wang et al., 2015) may be able to provide similar results with a parts-based matrix representation as opposed to PMF’s latent factor representation. Unlike PMF, which factors into only two matrices and captures a single layer of features, deep matrix factorization, inspired by the success of deep learning, aims to extract several features in a hierarchical way (Pierre and Siebert, 2020). Although many algorithms have been introduced for deep MF, it is still an emerging topic and questions of convergence, identifiability, and loss functions have not been fully explored. PMF on the contrary is well-explored algorithm with strong theoretical ground that has found success in a variety of matrix completion and collaborative filtering settings.

One limitation of PMF is that it suffers from the cold-start problem and is unable to predict the efficacy for compounds with no known values. Thus, any guided PMF assay must test at least one combination for every drug. It is also unknown how well PMF will scale to drug libraries larger than the ALMANAC. While our simulated experiment was more successful on the larger ALMANCAC data than the smaller Huntington’s disease data, PMF’s performance on larger drug libraries remains to be seen. We additionally note that both the ALMANAC and HD data sets employed combinations of compounds that were individually effective as monotherapies. To fully evaluate our method’s utility in guiding combination screens, we will need to apply it to screens in which not all compounds are individually efficacious. That is, the proper test of the method is its application to a new screen, which is beyond the scope of this work.

## Conclusion

We have shown that PMF can accurately impute missing values into the drug combination efficacy matrix for a screen, and that the performance of PMF does not depend on the efficacies of the drugs being tested. We further showed that PMF performs best when the input drug combination network has an Erdős-Réyni topology. Finally, we used simulated experiments to demonstrate that alternating PMF inference with experiments can efficiently identify the most efficacious two-drug combinations in a phenotypic screen.

There have been many other attempts at predicting the effects of drug combinations, and those that perform best include additional data, such as chemical structures, target profiles, or OMICS data (Wang et al., 2013; Huang et al., 2019; Menden et al., 2019). PMF has the advantage that its computation time scales linearly, and it can make accurate predictions for sparse and imbalanced data sets. Moreover, PMF is an unsupervised algorithm and by nature is easily interpretable as matrix factorizations easily provide a lens to determine relations, giving it several advantages over large deep learning networks. Our method is simpler by comparison, but it provides a baseline of performance against which more complicated prediction methods may be assessed. Indeed, not relying on additional information endows our method with flexibility: Instead of predicting the effects of combinations of drugs, it can be used to predict the effects of combinations of combinations, and we have no reason to believe that it will perform worse on unannotated compounds. On the contrary, our method may contribute to identifying mechanisms of action for novel compounds. The very ability of PMF to predict efficacies of combinations points to hidden mechanistic similarities within the set of compounds. By interpreting the PMF in terms of underlying biochemistry, we may gain insight into the nature of disease.

## Data Availability

The cancer datasets analyzed for this study can be found on the ALMANAC website (https://wiki.nci.nih.gov/display/NCIDTPdata/NCI-ALMANAC/). The HD data can be obtained from the authors. Source code is available online at https://github.com/lezonlab/pmf-drug-combo/.
